# Oxidative stress and its association with ST resolution and clinical outcome measures in patients with ST-segment elevation myocardial infarction (STEMI) undergoing primary percutaneous coronary intervention

**DOI:** 10.1186/s13104-020-05350-5

**Published:** 2020-11-11

**Authors:** Elmira Matin, Samad Ghaffari, Alireza Garjani, Neda Roshanravan, Somaieh Matin, Naimeh Mesri Alamdari, Naser Safaie

**Affiliations:** 1 Cardiovascular Research Center, Tabriz University of Medical Sciences, Tabriz, Iran; 2 Department of Pharmacology, School of Pharmacy, Tabriz University of Medical Sciences, Tabriz, Iran; 3 Department of Internal Medicine, School of Medicine, Ardabil University of Medical Sciences, Ardabil, Iran; 4 Department of Nutrition, School of Public Health, Iran University of Medical Sciences, Tehran, Iran

**Keywords:** Ischemia reperfusion injury, Oxidative stress, Myocardial infarction

## Abstract

**Objective:**

Reperfusion of ischemic myocardium generates oxidative stress, which itself can mediate myocardial injury. So, in this study, we investigated the level of oxidative stress markers and its association with clinical outcomes in patients with ST-segment elevation myocardial infarction (STEMI) undergoing primary percutaneous coronary intervention.

**Results:**

As indicated in the results, Post MI (Myocardial Infarction) heart failure was significantly higher in the group A (11% vs 4%, p = 0.047). Complete STR (ST-segment resolution) was observed to be significantly higher in the group B (36% vs 17%, p = 0.006). The SOD (Superoxide dismutase) and GPX (Glutathione peroxidase) levels were significantly higher in the group B compared to the other group (1547.51 ± 328.29 vs. 1449.97 ± 246.06, p = 0.019 and 60.62 ± 11.95 vs 57.41 ± 10.14, p = 0.042). The levels of GPX and SOD were shown to be directly related with complete STR and post PCI (Percutaneous coronary intervention)TIMI(Thrombolysis in Myocardial Infarction) flow 3 in the group A (p = 0.002 and p < 0.01, p = 0.005 and p < 0.02, respectively).

## Introduction

Cardiovascular disease is known as the leading cause of death worldwide [1]. Cardiovascular risk factors result in endothelial injury and propagate atherosclerosis [2]. Accordingly, acute myocardial infarction (MI) is one of the most prevalent forms of ischemic heart diseases [3]. The most important decision for a patient with ST-segment elevation myocardial infarction (STEMI) is the early and successful myocardial reperfusion by the use of thrombolytic therapy or primary percutaneous coronary intervention (PPCI), which has been shown as effective method on balancing the blood supply as well as demanding and improving the clinical outcome [4–6].

Various studies have indicated that, huge quantities of reactive oxygen species (ROS) are produced during reperfusion of the blood flow after MI [7, 8]. Consequently, these highly reactive species cause oxidative damage [9], which thereby lead to myocardial ischemia–reperfusion injury that can paradoxically reduce the beneficial effects of myocardial reperfusion [10, 11]. Malondialdehyde (MDA), as lipid peroxidation product, is known as one of the oxidative stress biomarkers [12, 13]. Beyond that superoxide dismutase (SOD) and glutathione peroxidase (GPX) are the first well- known line enzymes of the antioxidant defense [14, 15]. Correspondingly, some studies have shown the role of oxidant-antioxidant imbalance in the pathogenesis of myocardial ischemia and reperfusion injury. Moreover, antioxidant protects the cellular and sub-cellular membranes against the oxidative stress injury. It was indicated that the increased activity of total antioxidant capacity reduces the damages produced by the enhancement of lipid peroxidation, which may act by counteracting the harmful products [16, 17].

Thrombolysis in myocardial infarction (TIMI) flow is considered as the most valuable indicator of the epi cardial coronary flow. However, electrocardiographic ST resolution is a valuable marker of the improved myocardial reperfusion in microvascular system and also at the cellular level [18].

In this study, we investigated the probable role of stress oxidative markers (MDA, SOD, GPX, and Total antioxidant capacity (TAC)) among STEMI patients undergoing primary PCI based on Coronary TIMI flow, as well as their associations with in-hospital outcome. Therefore, in this study, we aimed to investigate the levels of MDA, SOD, GPX, and Total antioxidant capacity (TAC) as well as their relationships with ST resolution and clinical outcomes among STEMI patients undergoing primary PCI based on Coronary TIMI flow.

## Main text

### Methods

#### Methods and materials

This cross-sectional study was conducted in 2018on patients with STEMI in the first 6 h after their admission in the cardiology department at Madani Hospital, Tabriz, Iran.

The inclusion criteria were having resting chest pain for more than 20 min, showing variations in ECG with ST-segment elevations greater than 2 mm in precordial leads and greater than 1 mm in limb leads, and having serum troponin or CK-MB levels at least two-fold of the upper limit of normal range regarding the laboratory references.

**Fig. 1:**
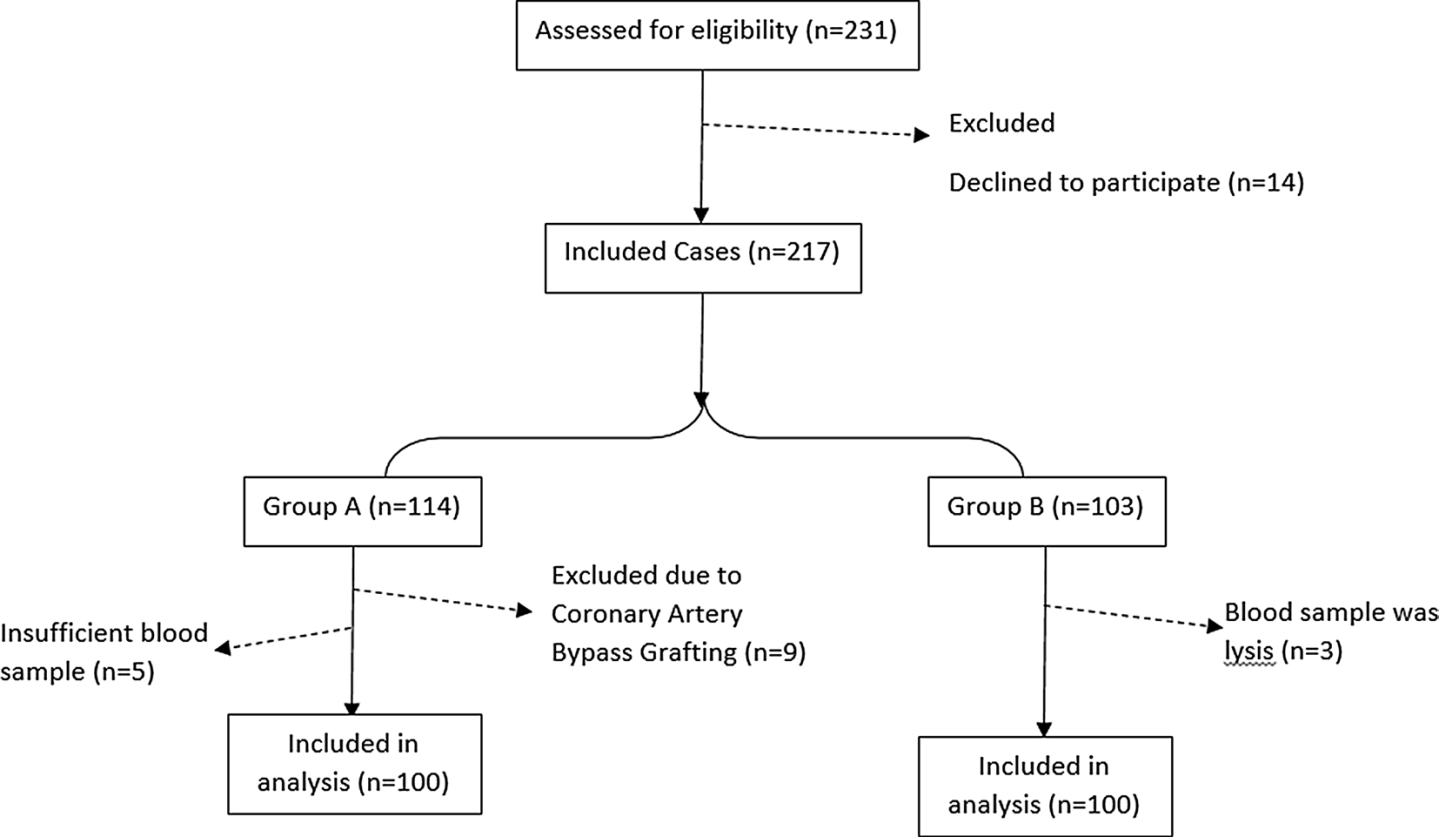
The flow diagram of the study and patients allocations in groups

The exclusion criteria were also as follows: having Left Bundle Branch Block, late presentation (> 6 h), using pace-maker, having the history of Heart failure or rescue PCI, chronic disease, malignancy, and being a candidate for emergency Coronary Artery Bypass Graft. Among the patients scheduled for primary PCI after matching with the study protocol, the first 100 consecutive patients with TIMI flow < 2 were enrolled in the group A and the first 100 patients with TIMI flow ≥ 2 were enrolled in the group B (Fig. 1). The written consent as well as a complete physical examination were obtained from the included patients. Electrocardiograms were recorded once on arrival and once 90 min after Primary PCI. STR (ST-segment resolution) was measured by passing 90 min from primary angioplasty at the same way to the maximal ST elevation in pre-angioplasty electrocardiogram. Mortality, arrhythmias, and post MI heart failure were investigated as hospital outcomes. Those patients with a Left ventricular ejection fraction (LVEF) < 40% at the time of discharge and rales in the lungs auscultation or congestion in Chest X-Ray (in terms of the American Heart Association guideline) [19] were considered as patients with heart failure. This study was ethically approved in the Research Department of Tabriz University of Medical Sciences (Iran) under the code of 60,109.

#### Biochemical information

After an overnight fasting of 12 h, five mL of venous blood samples were taken from each one of the participants. The obtained samples were centrifuged for 10 min at 1500 rpm to separate the serum and then kept at − 80 °C for MDA, SOD, GPX, and TAC tests. Afterward, RASOD kit (Randox, lab.crumlin.UK) and RANSEL kits (Randox, lab.crumlin.UK) were used for the assessment of SOD, GPX, TAC, and MDA levels [20].

#### Data analysis and statistical methods

The distribution of the data was checked using Kolmogorov- Smirnov test. The independent sample t-test was also used for comparing these two groups. Thereafter, Pearson coefficient correlation was used to evaluate the relationships among SOD, GPX, levels and the rate of ST-R. The results were then reported in the form of mean ± standard deviation. In this regard, IBM SPSS Statistics (ver.23) was applied to analyze all the obtained data. The significance level was considered as 0.05 for all of the tests.

### Results

In this study, 200 STEMI patients were investigated. Accordingly, no significant differences were found between these two groups regarding demographic and risk factors variables (Table 1).

**Table 1:** Comparison of demographic and baseline information and risk factors of CAD of Groups

Variable	Group A (n = 100)	Group B (n = 100)	p-value*
Age, years	60.01 ± 12.3	61.55 ± 11.32	p > 0.05 (NS)
Gender, male n (%)	80 (70)	73 (73)	p > 0.05 (NS)
Hypertension, n (%)	62 (62)	51 (51)	p > 0.05 (NS)
Diabetes mellitus, n (%)	33 (33)	24 (24)	p > 0.05 (NS)
Hyperlipidemia, n (%)	54 (54)	43 (43)	p > 0.05 (NS)
Smoking, n (%)	35 (35)	33 (33)	p > 0.05 (NS)
Family history, n (%)	32 (32)	27 (27)	p > 0.05 (NS)
Anterior STEMI, n (%)	61 (61)	56 (56)	p > 0.05 (NS)
Lateral STEMI, n (%)	5 (5)	6 (6)	p > 0.05 (NS)
Inferior RV STEMI, n (%)	9 (9)	15 (15)	p > 0.05 (NS)
Inferior STEMI, n (%)	25 (25)	23 (23)	p > 0.05 (NS)

NS, not significant, Group A: TIMI flow < 2, Group B: TIMI flow ≥ 2

*p-value was reported based on Chi-Square test & Independent sample t test

Moreover, there was no significant difference between the two groups regarding serum levels of MDA and TAC. However, SOD and GPX levels were also observed to be significantly higher in the group B as compared to the group A (Table 2).

**Table 2:** The average serum levels of biochemical factors in group A and B (mean ± SD)

Variable	Group A (n = 100)	Group B (n = 100)	p-value
MDA (nmol/ml)	2.33 ± 0.76	2.22 ± 0.82	0.133
TAC (mmol/l)	1.73 ± 0.4	1.65 ± 0.4	0.322
SOD (µ/g Hb)	1449.97 ± 246.06	1547.51 ± 328.29	0.019*
GPX (µ/g Hb)	57.41 ± 10.14	60.62 ± 11.95	0.042*

SD: standard deviation, SOD: superoxide dismutase, GPX: Glutathione Peroxidase, MDA: Malondialdehyde, TAC: Total anti-oxidant capacity

*p < 0.05 in based on independent samples *t*-test between two groups Group A: TIMI flow < 2, Group B: TIMI flow ≥ 2

ST segment resolution was divided into three categories as follows: I. no response to treatment (ST-R levels < 50%), II. partial response to the treatment (50% ≤ ST-R levels < 70%), and III. complete response to the treatment (ST-R levels ≥ 70%) [21]. By investigating the relationship among SOD and GPX levels and the rate of ST-R, it was revealed that in the group A, both of the variables were associated with the rate of ST-R, while in group B, only the level of GPX was related to the rate of ST- resolution (Additional file 1: Table S1).

These two groups were not significantly different in terms of death and arrhythmia. However, the difference between these two groups was turned out to be significant in terms of heart failure. The rate of complete ST-R was higher in the group B compared to that of the other group, which showed a significant difference between them. In addition, we observed a higher rate of TIMI 3 flow after PCI in the group B compared to group A (Additional file 1: Table S2).

### Discussion

The principal findings of this study were the higher level of SOD and GPX in patients with TIMI flow ≥ 2. Beyond that Post MI heart failure was significantly higher in patients with TIMI flow < 2. The higher level of complete STR in patients with TIMI flow ≥ 2 was other finding of this study.

The disruption in the blood flow and endothelial dysfunction, caused by the changes in the ion channels and the increased level of oxidative stress, may consequently lead to the formation of plaques in the coronary arteries [22]. In this regard, reactive oxygen species are considered as the main initiators of myocardial damages during reperfusion process [14]. Two of the antioxidant enzymes, named SOD and GPX play important roles in supporting and protecting cells against oxidative damages [23, 24]. Previous studies have indicated that in those patients with occluded arteries, due to the generation of reactive oxygen species that is the result of inflammations induced by tissue damages, primary PCI causes more damages [15]. Our study indicated that the serum levels of oxidative markers are significantly related to the severity of myocardial infarction and myocardial dysfunction. This finding is consistent with the findings of some previous studies [25, 26]. Furthermore, in this study, it was found that, the incidence rate of ventricular dysfunction was lower in the group with TIMI ≥ 2 compared to the other group. The main reason of this result might be the higher levels of endogenous antioxidant enzymes like SOD and GPX, which protect the heart against reperfusion-induced damages [25, 27].However, in our study, the two patients’ groups were not found to be significantly different in terms of the incidence of some complications such as arrhythmia and mortality.

One of the criteria that is considered in determining the level of response to treatment and the restoration of blood flow is ST-segment resolution [28]. Moreover, ST-segment resolution is a simple sign of myocardial reperfusion, which has been shown to be associated with left ventricular function recovery as well as mortality [14, 29]. In this regard, various studies have demonstrated that if ST-segment does not return to its normal condition up to 90 min after PCI, so it can be considered as a unfavorable prognosis [26, 30, 31]. Researchers in various studies have asserted that the lack of ST-segment resolution is the indicator of a perfusion defect in the myocardial tissue [29]. Our study have also indicated that the rate of complete ST-segment resolution to the baseline was lower in the patients of the TIMI < 2 group (STR ≥ 70%) compared to the patients in the TIMI ≥ 2 group, which can be explained by the induced myocardial dysfunction resulted from the generation of more free radicals [20].

In a previous study, Kammler et al. reported that, after PCI, the patients with TIMI flow of equal to 2 or less than 2 showed some adverse clinical complications during their hospitalization period as well as after 6-month follow-up from the time being discharged from the hospital [25]. Other studies have also indicated that the decreased blood supply by the coronary arteries after primary PCI is accompanied with adverse clinical outcomes [27, 32]. However, it should also be noted that some other factors such as age, clinical manifestations, comorbidities, and the location of myocardial infarction play critical roles in the final outcome of STEMI patients, as candidates for primary PCI [29, 31].

In the present study, lipid peroxidation was evaluated by measuring MDA level. In addition, the antioxidant activity was also assessed via measuring TAC [23]. No significant differences were observed between these two study groups in terms of their MDA and TAC levels. However, this issue requires further assessments in future studies.

In a study, it was observed that following Remote Ischemic Post Conditioning (RIPC) during primary PCI, the level of antioxidants has significantly increased, while the level of MDA has significantly decreased compared to the control group. Accordingly, these findings revealed the beneficial effects of RIPC on decreasing the reperfusion-induced damages [20].

The analysis of various studies revealed the fact that the relationships among the oxidative stress, antioxidant markers, and myocardial infarction are mixed. Moreover, some studies have indicated that high levels of these markers in patients are associated with myocardial infarction, while some others have rejected the existence of such associations [25, 28, 33]. Correspondingly, this might be due to the fact that the levels of oxidative stress markers depend on various factors such as the duration of the appearance of symptoms and comorbidities like diabetes, as well as the other chronic diseases [20, 23]. On the other hand, these biomarkers may be simply considered as a marker of severe injury, so performing further studies by matching their levels with the enzymatic infarct size or cardiac MR findings may be helpful to test this hypothesis.

### Conclusion

The findings of this study indicated that antioxidant enzymes levels in patients with STEMI are significantly associated with coronary artery stenosis and the level of responses to treatment. However, such relationships were not observed regarding the TAC and MDA levels. The imbalance between the production of free radicals and antioxidant defense systems was shown to be strongly associated with cardiovascular disease and its unfortunate outcome.

## Limitations

Considering the limitations of the present study in measuring the levels of all antioxidant substances (selenium, vitamin C, etc.), it seems that performing further studies is necessary. Future studies with large sample size and by parallel measuring the levels of other antioxidant substances and some inflammatory factors like hs-CRP, might be helpful for having more definitive conclusions.

## Supplementary information

**Additional file 1: Table S1.** Comparison SOD and GPX levels and the rate of ST-R between the two groups.** Table S2**. Hospital outcomes, complete response to treatment and post PCI TIMI.

## Data Availability

The datasets used and/or analyzed during the current study are available from the corresponding author on reasonable request.

## Abbreviations

MI: Acute myocardial infarction
STEMI: ST-segment elevation myocardial infarction
PPCI: Primary percutaneous coronary intervention
ROS: Reactive Oxygen Species
MDA: Malondialdehyde
SOD: Superoxide dismutase
GPX: Glutathione peroxidase
TAC: Total antioxidant capacity
TIMI flow: Thrombolysis in myocardial infarction flow
LVEF: Left ventricular ejection fraction
hs-CRP: High sensitive C Reactive Protein
