# Supplementary material for: Oxidative stress and its association with ST resolution and clinical outcome measures in patients with ST-segment elevation myocardial infarction (STEMI) undergoing primary percutaneous coronary intervention
Source: BMC Res Notes. 2020 Nov 11;13:525. doi: 10.1186/s13104-020-05350-5 (PMC7656688; doi:10.1186/s13104-020-05350-5)
Supplement: Supplementary file 1 — Additional file 1: Table S1. Comparison SOD and GPX levels and the rate of ST-R between the two groups. Table S2. Hospital outcomes, complete response to treatment and post PCI TIMI. [file 13104_2020_5350_MOESM1_ESM.docx]

**Table S1: Comparison SOD and GPX levels and the rate of ST-R between the two groups**

|  | P-value | | III  (STR≥ 70%) | II  (50%≤ STR< 70%) | I  (STR < 50%) |  |  | Group |
| --- | --- | --- | --- | --- | --- | --- | --- | --- |
|  | 0.002*, r=0.81 | | 63.02±8.72 | 56.03±10.14 | 53.08±8.9 | GPX(u/g Hb) |  | A |
|  | 0.000*, r=0.72 | | 1582.44±188.25 | 1451.05±240.11 | 1236±201.57 | SOD(u/g Hb) |  |  |
|  | 0.023*, r=0.60 | | 63.86±9.76 | 58.5±13.11 | 55.67±10.5 | GPX(u/g Hb) |  | B |
|  | 0.871,  r=0 | | 1523.1±201.70 | 1565.42±384.64 | 1534.98±298.37 | SOD(u/g Hb) |  |  |
|  |  | Abbreviation: SD: standard deviation, TIMI: Thrombolysis in myocardial infarction, STR: ST - Resolution, SOD: superoxide dismutase, GPX: Glutathione Peroxidase, MDA: Malondialdehyde, TAC: Total anti-oxidant. I: ST-R levels < 50%, II: 50%≤ ST-R levels< 70%, III: ST-R levels ≥ 70%. Notes:^*^*p*<0.05 is based on in Pearson Correlation. Group A: TIMI flow< 2, Group B: TIMI flow≥2 | | | | | | |

**Table S2: Hospital outcomes, complete response to treatment and post PCI TIMI**

| p-value | Group B  (n=100) | Group A  (n=100) | Variable |
| --- | --- | --- | --- |
| 0.047*  0.155  0.366 | 4%  0%  10% | 11%  2%  12% | In Hospital outcome   - Heart Failure, n (%) - Death, n (%) - Arrhythmia (Sustained VT/VF), n (%) |
| 0.171 | 13% | 18% | (HF or arrhythmia) |
| 0.045* | 14% | 23% | (HF or Death or arrhythmia) |
| 0.005* | 100% | 88% | TIMI3 patency after PCI |
| 0.006* | 36% | 17% | Complete ST-segment  resolution (≥70%) at 90min (post PCI) |
| Abbreviation: SD: standard deviation, TIMI :Thrombolysis in myocardial infarction ,PCI: percutaneous coronary intervention, MI :myocardial infarction, VT: Ventricular Tachycardia, VF: Ventricular Fibrillation .Notes:^*^*p*<0.05 is based on independent samples *t*-test between two groups Group A: TIMI flow< 2, Group B: TIMI flow≥2 | | | |
